# Physiological Basis of Sex Differences in Human Performance and Exercise‐Associated Pathology

**DOI:** 10.1111/cen.70150

**Published:** 2026-04-29

**Authors:** David A. Holdsworth, Thomas J. O'Leary, Craig L. Doig, Natalie Taylor, Jasminder Dosanjh, David R. Woods, Michael J. Stacey, Robert M. Gifford

**Affiliations:** ^1^ Academic Department of Military Medicine, Research and Clinical Innovation Royal Centre of Defence Medicine Birmingham UK; ^2^ Radcliffe Department of Medicine, Acute Multidisciplinary Imaging and Interventional Centre, Division of Cardiovascular Medicine University of Oxford Oxford UK; ^3^ Army Health and Performance Research Army Health Branch, Army Headquarters Andover UK; ^4^ Division of Surgery and Interventional Science University College London London UK; ^5^ Department of Biosciences, Centre for Systems Health and Integrated Metabolic Research Nottingham Trent University Nottingham UK; ^6^ Carnegie Institute of Sports Science Leeds Beckett University Leeds UK; ^7^ Department of Surgery of Cancer Imperial College London London UK; ^8^ Institute for Neuroscience and Cardiovascular Research University of Edinburgh Edinburgh UK

**Keywords:** cardiovascular differences in endurance, endocrine physiology, exercise‐associated pathology, sex differences in performance, testosterone and physical performance

## Abstract

The presence of sex differences in human physical performance is well‐established and shaped by distinct endocrine, anatomical and physiological mechanisms. Despite sustained advances, our understanding of how inherent biological factors drive variations in exercise capacity and related pathologies is still developing. This review examines data primarily from human trials on hormonal and other physiological determinants of sex differences in exercise performance. Higher testosterone in men leads to greater muscle mass, haemoglobin, bone size and cardiac output, explaining much of the performance gap between sexes. Female physiology confers advantages during prolonged submaximal exercise, including greater fat oxidation, lower carbohydrate dependence and improved fatigue resistance, which may help explain narrower gaps in ultra‐endurance events. These differences translate into a persistent performance gap (around 10%–15%) in most sports, particularly those requiring strength and power, although the gap narrows as event duration increases. Sex differences also influence responses to training and risk of exercise‐related pathology, including differences in stress responses, reproductive dysfunction (e.g., hypothalamic amenorrhoea), musculoskeletal injury and thermoregulation. These insights inform specific approaches in sports medicine and clinical endocrinology, emphasising the importance of sex as a fundamental biological variable in exercise physiology, sports performance and in clinical management of athletes and those in physically demanding jobs.

## 1 Introduction

Increased female participation in sport and physically demanding occupations has driven the need to understand sex‐specific physiology and exercise‐associated pathology in recent decades. Women are entering environments historically studied almost exclusively in men, including elite sport, military training and extreme endurance events. Early improvements in female performance largely reflected expanding opportunities for participation; however, now that representation is approaching parity, biological determinants of differences in performance are becoming increasingly apparent. Gender reassignment therapies have shifted traditional boundaries for sports participation, prompting debate about the rationale for sex‐based demarcation. The SRY gene, which triggers male gonadal development, is now tested by World Athletics to distinguish male and female categories. While this has not ended all controversy on the matter [1], it underlines the importance of understanding endocrine physiology as the basis of sex differences in physical performance.

While many previous reviews have focused on physiological and morphological differences contributing to the sex differences in human performance, few have characterised endocrinological factors which underpin these differences. In many settings of exercise, physiological stressors may interact with endocrine biology to influence both performance and health outcomes. It follows that an appreciation of sex as a biological variable in physiology, particularly gonadal hormones, is necessary to understand the distinction between the sexes in physical performance and exercise‐related pathology. Understanding sex differences improves performance optimisation, injury prevention, clinical care and policy decisions in sport, health and physically demanding occupations.

This review aims to describe the endocrine basis of sex differences in performance, with a focus on how these shape cardiovascular and muscular adaptation, metabolic substrate utilisation and thermoregulation. We then consider how these physiological differences influence patterns of exercise‐associated pathology. These are intended to provide clinicians and sports scientists with an endocrine framework for understanding the interplay between biological sex, exercise performance and health outcomes.

## 2 Historical Trends in Physical Performance

In the 55 years since women were first permitted to compete over a marathon distance, the women's record improved by 25% while the men's record improved by < 5%. The disparity in the rate of improvement was even greater in swimming. In 1977, based on the rate of closure of the sex gap in performance, it was predicted that women would catch up with men in the first decade of the 21st century [2].

The majority of the improvement in female performance occurred in the early decades of female competition and has been attributed to the sociocultural phenomenon of opening up women's sport [1]. While women took no part in the first modern Olympics of 1896 in Athens, by Paris 2024, there was parity between female and male representation [2]. The marathon itself was not open to female Olympians until 1984; over 11 intervening games, the sex difference in performance has been inversely proportional to the ratio of female to male competitors [2]. As more women participate, the talent pool increases.

As equality of participation is addressed, biological factors underpinning sex differences become more apparent (Table 1). A performance gap persists in most disciplines, with men maintaining a consistent lead over women in strength and power‐based events; however, the gap tends to narrow as the distance and duration lengthen. Likewise, in swimming, the gap narrows with increasing distance and in ultra‐distance or cold‐water events, women often match or outperform men [2]. When considering group averages in long‐distance swimming rather than individual fastest times, women swim faster than men in any given event and may also outperform men in colder water [8].

**Table 1:** Sex differences in current world records.

Event	Men	Women	Sex difference (%)
Weightlifting (kg)			
Heaviest lifters (M ≥ 105 kg; F ≥ 90 kg) Snatch	220	155	29.5
Clean and Jerk	263	193	26.6
Total	477	348	27.0
56–58 kg category (M56 kg; F58 kg) Snatch	139	112	19.4
Clean and Jerk	171	142	17.0
Total	307	252	17.9
Jumping (m)			
Pole vault	6.29	5.06	19.6
Long jump	8.95	7.55	15.6
Triple jump	18.29	15.74	13.9
High jump	2.37	2.10	11.4
Running			
60 m (s)	6.34	6.92	9.10
100 m (s)	9.58	10.49	9.50
200 m (s)	19.19	21.34	11.2
400 m (s)	43.03	47.6	10.6
800 m (min:s)	1:40.91	1:53.28	12.3
1500 m (min:s)	3:26.00	3:48.68	11.0
5000 m (min:s)	12:35.36	13:58.06	10.9
10,000 m (min:s)	26:11.00	28:54.14	10.4
Marathon, 42.195 km (h:mm:ss)	2:00:35	2:09:56	7.80
Swimming			
50 m (s)	20.91	23.61	12.9
100 m (s)	46.40	51.71	11.4
200 m (min:s)	01:42.00	01:52.23	10.0
400 m (min:s)	03:39.96	03:54.18	6.5
800 m (min:s)	07:32.12	08:04.12	7.1
1500 m (min:s)	14:30.67	15:20.48	5.7
10,000 m (h:min:s)	1:48:33.7	1:59:30.9	10.1
English Channel (h:min:s)	07:17	07:40*	5.3
Catalina Channel (h:min:s)	07:37:31	07:15:55*	−4.7
Manhattan Island Race (h:min:s)	05:34:58	05:44:03	2.7

*Note:* Data as of September 2025, except where noted. Sex difference calculated as [Δ men:women]/men*100. *records held by Penny Lee Dean (Catalina 1976, English Channel 1978). The International Weightlifting Foundation (IWF) records up to 2018 are used, because the categories were altered in 2019, and the IWF ‘World Standard’ has not yet been achieved in an accredited lifting event across the majority of events [3, 4, 5, 6, 7].

### 2.1 Sex Steroids Are Key Determinants of Physical Performance

Sex differences in physicality are evident from an early age. After minipuberty, boys demonstrate greater lean mass, less adiposity and faster average run times than girls [9], although this is only apparent in swimming after age 10 [10]. Testosterone levels are generally 15–20 times higher in men than women after puberty. In 2018, the International Amateur Athletics Federation (now World Athletics) stipulated a testosterone cutoff of 5 nmol/L or less (in androgen‐sensitive individuals) as a criterion for participation in the female classification.

Testosterone exerts genomic effects in skeletal muscle, bone and bone marrow through binding androgen receptor (AR) and as a prohormone of 17*β*‐oestradiol and 5*⍺*‐dihydrotestosterone [11]. Testosterone wields strong anabolic effects on skeletal muscle protein synthesis, satellite cell content and capillarisation and skeletal muscle cross‐sectional area, by driving the development of both type I and II muscle fibres [12]. Muscle power is the product of contractile force and velocity; the shortening velocity of type II fibres is up to double that of type I fibres. Since male muscle has a higher proportion of type II fibres, and a higher total area of type II fibres in absolute terms, both the contractile force and velocity are higher than in female muscle, resulting in greater power [13]. Although men and women demonstrate similar relative improvements in muscle strength and hypertrophy following resistance training, absolute gains are typically greater in men, although at repeated contractions at the same relative intensity, women show greater resistance to muscle fatigue [14]. Testosterone also exerts anticatabolic effects by downregulating glucocorticoid receptor expression in skeletal muscle, reducing proteolysis [15].

Testosterone increases haemoglobin mass by a variety of mechanisms. Testosterone−AR complex stimulates renal erythropoietin production and increases iron availability by inhibiting hepcidin, and increases the production of myeloid progenitor cells, mediating an adjustment in the set‐point for haemoglobin in adults. The haemoglobin concentration is 12% lower in pre‐menopausal women than in men of the same age and race [16]. The effect of female sex steroids in promoting fluid retention are insufficient to compensate for the reduced blood volume [17]. The key differentiator is higher haemoglobin mass in men, derived from higher testosterone relative to 17*β*‐oestradiol [16].

Testosterone−AR complex directly stimulates trabecular bone formation and skeletal muscle mass indirectly influences remodelling, increasing force‐derived bone mass and strength [18]. However, the principal means by which testosterone increases bone density and strength is via aromatisation to 17*β*‐oestradiol, which inhibits osteoclasts, stimulates osteoblast activity and promotes osteocyte survival, reducing expression of sclerostin [18].

Concentrations of 17*β*‐oestradiol and progesterone are normally significantly higher among women, yet approach male levels in the early follicular phase [13]. For this reason, many exercise studies comparing women with men are conducted during the follicular phase. 17*β*‐oestradiol exerts genomic effects via oestrogen receptors (ER)α and β. ERα, the predominant isoform in skeletal muscle, enhances lipolysis, fat oxidation and insulin sensitivity [19]. After menopause, methylation of the promoter region of the ERα gene leads to predominance of ERβ, promoting lipogenesis and insulin resistance [20]. Hypothalamic ERα stimulation decreases the threshold for arginine vasopressin (AVP), increasing AVP‐dependent water retention and plasma volume [21].

Progesterone decreases skeletal muscle insulin sensitivity, increases hepatic lipid content and may suppress gluconeogenesis during exercise [20]. High progesterone concentrations in the luteal phase increase core temperature by 0.5°C and raise the threshold for peripheral vasodilation and sweating [22]. Progesterone also contributes to increased plasma volume, increasing overall extracellular volume and perhaps oncotic pressure, by reducing albumin transcapillary escape [21].

### 2.2 The Hypothalamic−Pituitary−Adrenal (HPA) Axis Response to Exercise

The HPA is a critical regulator of homoeostasis and integrator of stress responses. Through the release of glucocorticoids, it acts to drive appropriate energetic decision‐making processes such as substrate utilisation and lipid storage. Human males tend to exhibit a greater increase in cortisol levels than females in response to stress [23]. However, the type of stressor and the age of the individual can impact the magnitude of response, as can psychosocial variables [24, 25, 26]. Gonadal hormones also play an important role in conferring sex differences in the HPA axis response to stress, fundamentally shaping how males and females respond to physical activity [27]. A recent review highlighted sexual dimorphism in glucocorticoid functionality and the formation of diseases associated with HPA axis dysfunction, suggesting new underlying concepts and causal mechanisms [28]. HPA activation appears to interact with biological sex, with considerable complexity. As an example, following psychological stress, adrenocorticotrophin (ACTH) and cortisol responses were significantly greater in male subjects compared to female subjects, while cortisol responses to pharmacological challenges were significantly greater in females [29].

A recent study examining prolonged physical stress provided valuable insight into sex‐specific adaptation. Over 11 months of military training, sex‐related differences emerged in both hypothalamic−pituitary−gonadal (HPG) and HPA axis response, with HPA axis response to intensive training being greater among women than men [30]. This suggests females may experience more pronounced HPA axis activation during sustained periods of intense physical activity. Determinants of this response, oestrogen or otherwise, remain to be identified.

HPA axis regulation by exercise has been demonstrated, with studies showing that physical activity can modify stress responsivity of the HPA axis [31]. Importantly, chronic exercise training attenuates HPA axis reactivity across both sexes. Studies examining HPA axis reactivity and autonomic response to acute stress have shown that training can modify how the HPA axis responds to subsequent stressors [32]. These data suggest regular physical activity may be protective against excessive stress reactivity. However, this study grouped males and females together. Increased appreciation of biological sex as a variable is better defining the responses in males and females; in turn, this is improving our understanding of how the HPA determines responses to physical activity.

Thus, the HPA axis shows sexual dimorphism in its response to physical activity. These responses have important performance and clinical implications for developing appropriate training protocols and preventing deleterious responses in both males and females. A greater HPA axis activation observed in females during intense training periods suggests that monitoring stress biomarkers could be particularly important for females to prevent maladaptive responses and optimise performance and sustain health.

### 2.3 Cardiovascular Differences Are Critical to Differences in Endurance Performance

**Figure 1:**
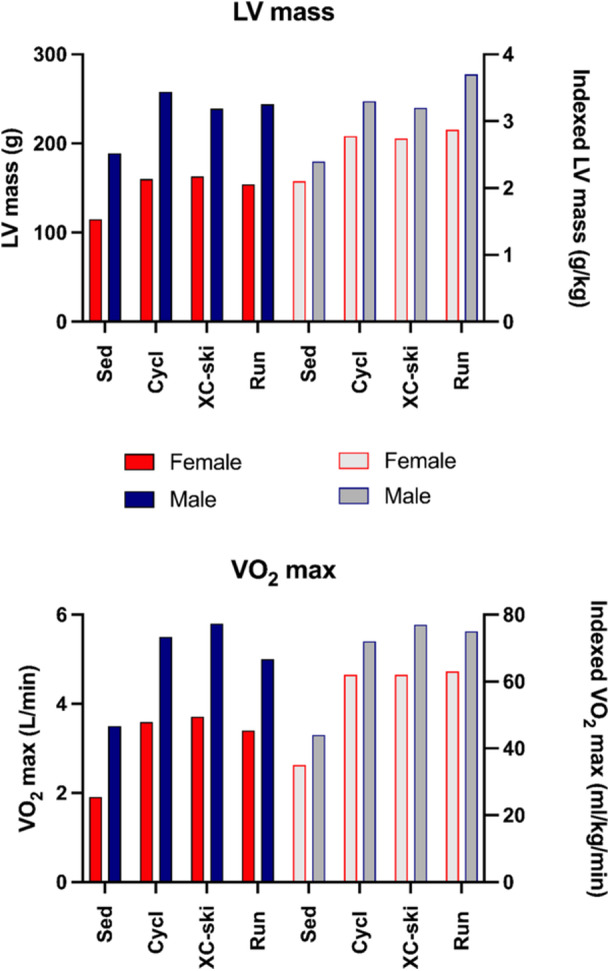
Sex differences in absolute and weight‐adjusted LV mass and maximal oxygen uptake (V̇O_2_max). Figure adapted from Riley‐Hagan et al. [35] and Milliken et al. [36]. Cycl, trained cyclists; Run, trained runners; Sed, healthy sedentary controls; XC‐ski, trained cross‐country skiers.

It is widely recognised that endurance performance can be predicted by maximal oxygen uptake (V̇O_2_ max), proportion of V̇O_2_ max that can be sustained (≈ % V̇O_2_ max at the anaerobic threshold) and economy of movement. Endurance exercise is limited by cardiac output (the product of stroke volume and heart rate), total body haemoglobin, muscle blood flow and muscle oxygen utilisation [33]. Of these, the factor which responds most significantly and rapidly to training and detraining is stroke volume [34]. While the male heart is larger from puberty onwards, with increased wall thickness and larger chamber volumes, the female heart is *not* just a small male heart. Cardiac mass, cardiac chamber dimensions and stroke volume are greater in men in absolute terms *and* when normalised to body mass, body surface area and lean mass [35]. These observations explain the higher maximum oxygen uptake than women across a range of sports (Figure 1) [37]. There are also important sex differences in relative dimensions of the left and right heart, autonomic inputs, calcium handling, myocyte action potential (slower repolarisation in females leading to a longer QT interval) and in cardiac adaptation to physical training. The degree of exercise adaptation to increase stroke volume in women is generally less, with a plateau occurring earlier (3−6 months), than in men (≈ 12 months) [13].

There is a considerable body of evidence supporting the role of sex hormones in the mechanisms underlying sex differences in stroke volume. 17*β*‐oestradiol reduces the vasomotor response to noradrenaline and increases nitric oxide levels during exercise [37]. These effects combine to increase the volume of distribution for the blood, reducing venous return, end‐diastolic volume and consequently both cardiac contractility and stroke volume. More direct evidence comes from murine data, where oestrogens directly downregulate L‐type calcium channels, lowering intracellular calcium flux and myocyte contractility [38]. This also offers a potential explanation for the increased prevalence of ventricular arrhythmia in men. Exogenous androgens increase, whilst orchidectomy reduces, cardiac muscle mass and contractility in female and male rats [39]. Observations from anabolic‐androgenic steroid users corroborate these findings in humans [40].

The smaller size of the female heart is associated with a different physiological mechanism to augment cardiac output. For a given ‘metabolic load’ standardised by % V̇O_2_max, cardiac output does not differ between the sexes, but the increased flow is delivered through a faster heart rate at lower stroke volume [41]. For any given workload, the necessary cardiac output is higher in women than in men, again delivered through a smaller stroke volume at a higher heart rate.

Studies of sex differences in cardiovascular autonomic activity and the cardiac action potential reveal increased parasympathetic tone in women and increased sympathetic tone in men; decreased sensitivity of the baroreflex response (a short term [seconds] reflex reduction in heart rate in response to increased arterial pressure and a longer term [minutes] reduction in peripheral vascular tone) in women and prolonged repolarization time, mediated (in animal models) by a reduced potassium current in females [42]. Men generally experience a larger increase in systolic blood pressure than women, driven by higher catecholamine concentrations, while women often show a greater increase in heart rate and greater cerebrovascular vasodilation [42]. 17β‐oestradiol reduces the occurrence of catecholamine‐induced endothelial dysfunction by enhancing nitric oxide production and attenuating the reactivity of β2 adrenergic receptors [37].

When absolute measurements of cardiac output and stroke volume are normalised to lean mass (compensating for the fact women are normally smaller than men and have higher proportionate fat mass) this reduces, but does not abolish, the sex differences [36]. Even with such lean mass normalisation, the significant sex differences in haemoglobin and blood volume persist [43, 44]. A combination of these effects on cardiac output and oxygen carrying capacity are likely to account for most of the 10%−15% lower peak oxygen uptake values in women [2].

### 2.4 Differences in Muscle Biochemistry and Substrate Utilisation Make Female Physiology Better Suited to Prolonged Submaximal Exercise

At a whole body level, the respiratory exchange ratio (the ratio of CO_2_ evolved and O_2_ consumed, measured at the mouth) is lower in women than in men at any given exercise intensity. This finding has been demonstrated in meta‐analyses of multiple studies [45] and reflects a greater whole‐body predominance of carbohydrate over fat oxidation in men over a range of exercise intensities [46]. The fact that women consistently show a higher ratio of fat to carbohydrate oxidation both before and after supervised exercise training suggests this is an inherent, sex‐based difference rather than a result of training status [47]. The higher proportion of Type 1 (‘slow twitch’) muscle fibres in women undoubtedly contributes to the finding of higher fat oxidation [48]; however, administration of exogenous 17*β*‐oestradiol shifts substrate preference towards fat oxidation, without altering fibre type proportion, in men [49]. Female skeletal muscle contains more intramyocellular lipid (IMCL) than male, and the rate of IMCL consumption is higher in women than men during 90 min submaximal exercise [50]. The higher rate of glycerol appearance in blood seen in women during submaximal exercise suggests that there is not only an increased supply of available fat from within the muscle stores, but also from mobilisation of body fat depots [47]. Most [51, 52, 53, 54], though not all [55], studies have demonstrated a higher rate of fat oxidation in women during the mid‐luteal than mid‐follicular phase. The rate of glycerol appearance (representing peripheral lipolysis) is also increased during the mid‐luteal stage of the menstrual cycle [55]. While significant, the effect sizes seen are small. Consistent with the finding of higher rates of fat oxidation in women, mRNA transcripts from skeletal muscle genes for the mobilisation (lipolysis), transport and oxidation of fat are higher in women than men, and have been demonstrated to rise in men with acute administration of 17*β*‐oestradiol [45, 49].

Conversely, evidence suggests that females exhibit reduced carbohydrate oxidation compared to males. In the rat, 17‐β‐oestradiol administration reduces glycogen consumption in heart and skeletal muscle [56], while surgical resection of the ovaries increases skeletal muscle glycogen consumption during prolonged exercise [57]. In exercising women, in whom endogenous sex hormones have been suppressed, the exogenous administration of 17*β*‐oestradiol reduces the rate of gluconeogenesis and glycogenolysis back to the baseline condition [58]. In contrast to the concordance of higher mRNA products of the genes of lipid metabolism in women with higher rates of fat oxidation, and the reproduction of many of these higher mRNA levels in men after receiving exogenous 17‐β‐oestradiol, no such concordance has been seen in the sex pattern of the gene transcripts of carbohydrate metabolism [45, 49, 59]. The most plausible explanation for this is that the sex difference in rates of carbohydrate oxidation is a secondary outcome of the difference in fat oxidation. As described in the seminal work of Randle et al., there is direct and indirect suppression of glycolysis in the presence of abundant fat [60].

The decreased female dependency on carbohydrate utilisation during prolonged exercise offers a potential explanation for the decrease in the sex difference in performance over increasingly longer duration events. In their seminal work in the 1960s, Erik Hultman and Jonas Bergstrom demonstrated the critical importance of skeletal muscle glycogen in the aetiology of muscular fatigue and a critical limiter of endurance capacity [61]. The critical problem with reaching a point of glycogen depletion in skeletal muscle is not that there is no carbohydrate available for oxidation (fat remains in abundant supply as a fuel supply), but that there is no carbohydrate available to replenish the intermediates of the TCA cycle (it is the furnace which runs out rather than the fuel). The pathways of replenishment, ‘anaplerotic pathways’ include the conversion of the substrates of terminal glycolysis (phosphoenolpyruvate and pyruvate) into the 4‐carbon intermediates of the TCA cycle (malate and oxaloacetate). This process, which depends upon locally stored glycogen, continually regenerates the TCA cycle and maintains a supply of ‘Acetyl‐CoA acceptors’ to sustain the ‘oxidative capacity’ of the mitochondrion [62].

Sex differences in protein metabolism may account for superior fatigue resistance in prolonged, submaximal exercise among women [63]. During endurance training, women consistently excrete proportionally less nitrogen than men, reflecting lower skeletal muscle turnover [64]. This relates in large part to a reduced oxidation of leucine in women, which in turn is associated with decreased activity of the branched‐chain 2‐oxo acid dehydrogenase [65]. In an elegant blinded, randomised study, Hamadeh et al. measured the effect of 17*β*‐oestradiol on 12 men over 8 days. During 90 min bouts of cycling, the men treated with oestradiol consumed 16% less leucine [66]. This was associated with 44% increase in fat oxidation and a reduction in carbohydrate oxidation of 16%. Sex differences in pacing strategies may also contribute to reduced fatiguability among women.

### 2.5 Sex Differences Persist in Ultra‐Endurance Performance

Women seem to have fewer physiological disadvantages in ultra‐endurance exercise—benefiting from the so‐called ‘oestrogen advantage’ in metabolism and resistance to fatigue. Women have demonstrated excellent physical performance and resilient endocrine function during submaximal exercise sustained for weeks or months [67]. However, this advantage has yet to be fully reflected in outcomes of ultra‐endurance races. For example, fixed‐distance ultramarathons (Table 2) demonstrate a greater sex‐difference performance than expected from trends in long‐distance running (Table 1). In a review of time‐limited ultramarathons (where athletes run as far as possible in a fixed time, e.g., 6, 12 or 24 h), women were shown to have marginally closed the gap. However, this was partly because the age of male participants increased more than that of female participants [72].

**Table 2:** Course record times at leading mountain ultramarathons.

**Ultramarathon** (distance, ascent)	**Men**	**Women**	**Sex difference (%)**
UTMB OCC (56 km + 3460 m)	5:00:35	5:56:04	18.5
UTMB CCC (101 km + 6100 m)	10:14:25	11:41:55	14.2
UTMB (171 km + 10,040 m)	19:37:43	22:09:31	12.9
Tor de Géants (330 km + 24,000 m)	66:08:22	79:10:40	19.7
Marathon de Sables (typically 250 km + 3500 m)	16:22:29	21:35:22	31.8
Hardrock 100 (165 km, +10,000 m)	21:33:06	25:50:23	19.9

*Note:* Data correct in March 2026 [68, 69, 70, 71]. Times given in hh:mm:ss. Sex difference calculated as [Δ men:women]/men*100.

Abbreviations: CCC, Courmayeur–Champex–Chamonix; OCC, Orsières–Champex–Chamonix; UTMB, Ultra Trail du Mont Blanc.

Important considerations to interpreting results from such events include lower female representation, psychological differences, a general financial barrier to representation for many events (due to high entrance fees) and, crucially, fuelling: participants are provided carbohydrate‐rich fluids and/or foods at regular intervals, eliminating any advantage of lipid substrate delivery conferred by 17*β*‐oestradiol. The discrepancy potential (biological advantage) and expression (outcomes) continue to be shaped by training opportunity, participation rates, cultural or logistical barriers or design of event formats, which may make them better suited to male physiology. Box 1 presents a personal reflection on the relationship between the expected narrowing of sex differences in performance and the lived reality of a female ultrarunner.

Box 1: **‘Closing the gap?’ The experience of a female professional ultrarunner.**

Having competed in ultramarathons for over a decade, I have gone from a ‘back of the pack’ runner to competing for podiums. With focused and consistent training, improved nutrition and a wealth of scientific data to drive individuals to optimise performance, the gap between men and women still exists. Courtney Dauwalter, arguably the most successful female ultra‐runner in the last decade, has won races outright, beating the male competition. But when racing against the top of the men's field, she will come up short; the course records for men and women for UTMB evidence this.

Although there is an ‘oestrogen advantage’ for women during endurance events, the psychological and logistical elements of racing are areas that both men and women are focusing some training time on and seem increasingly important for success. I suspect that when women were first allowed to compete in the Olympics and female times fell rapidly, there was an element of increased participation and focused training. Thus, in the forthcoming decade, we will likely see men and women break records in ultrarunning as a result of better race preparation, including refining these non‐physiological elements of endurance sport that are essential to success and performance. Most coaches today spend time with their athletes working on the psychological and logistical preparation, as well as the physical aspect. Ultrarunning is becoming a team sport with racers having a support crew at aid stations supporting a runner to pack appropriate nutrition, sleep strategy, clothing and safety gear as efficiently as possible, so reducing time spent static in an aid station, and the time taken to complete a race. These small improvements, regardless of sex, will impact results and may explain why there is a discordance between the expected female advantage and persistent sex differences in ultra‐marathons.3. Sex Differences in Exercise‐Associated Pathology.

## 3 Sex Differences in Exercise‐Associated Pathology

Exercise physiology and performance discrepancies invite a broader discussion of how sex differences manifest in athletes within endocrinology. It has been seen that biological factors including sex steroids, HPA axis activity, substrate utilisation and muscle fibre composition, shape sex differences in strength and endurance. These factors also may influence vulnerability to exercise‐associated pathophysiology, such as reproductive dysfunction [73], relative energy deficiency [74], musculoskeletal injury [75] and heat illness [76].

### 3.1 Reproductive and HPA Axis Dysfunction: Relative Energy Deficiency and Overtraining Syndrome (OTS)

Most evidence of reproductive dysfunction in athletes has originated from women. Ascertainment bias is likely to partly explain this: suppression of the HPG axis becomes clinically apparent much earlier in women than in men. HPG axis function [77] and bone health [78] are reversibly disrupted from low energy intake during exercise, via HPA axis upregulation [79]. The ‘female athlete triad’ comprises insufficient energy availability, oligomenorrhoea and osteopenia: if residual caloric energy after locomotion is insufficient, reproductive function and bone turnover cannot be maintained [80]. This paradigm was expanded into the Relative Energy Deficiency in sports (REDs) paradigm, which attributes a wide array of clinical problems to energy deficiency after exercise in men and women, including immune and haematological dysfunction, disordered sleep and mental illness [74]. The limited data supporting REDs in men suggest they require a greater energy deficit to develop hypogonadism [81]. The paradigm will be appreciated by those caring for patients with recognisable energy deficit, for example, athletes undertaking high volume exercise with cognitive dietary restraint, perfectionist traits or a drive for thinness, or those with frank eating disorders.

The REDs model has attracted criticism because it ascribes several complex pathologies to a single aetiology [82]. An overlapping and sometimes competing model of exercise‐associated hypogonadism is OTS, caused by excessive training without sufficient recovery (‘over‐reaching’) [83]. OTS typically presents with persistent fatigue, risk of injury and declining performance, frequently occurring in the context of other stressors like poor sleep or psychosocial stress. Both REDs and OTS share upregulation of the HPA axis as a final common pathway, suppressing release of gonadotropin‐releasing hormone (GnRH), luteinising hormone (LH) and follicle‐stimulating hormone (FSH). Both are associated with hypothalamic amenorrhoea, or male hypogonadism; however, OTS appears to affect men and women with greater parity than REDs. Quite marked upregulation of the HPA axis has been observed in OTS [84], perhaps reflecting the wider array or greater acuity of underlying causal stressors.

Men and women demonstrate HPG axis suppression when high volumes of exercise are conducted alongside multiple non‐exercise stressors, as occurs during military training [85]. During exposure to basic military training, female HPA and HPG appear to demonstrate greater disruption than males [30]. However, during prolonged, highly arduous training associated with an energy deficit, female reproductive function is more resilient [67, 86]. In this setting, the physiological advantages of 17*β*‐oestradiol for substrate utilisation and fatiguability, and perhaps nontechnical factors, may become especially relevant.

It has been suggested that OTS can only be diagnosed when energy deficiency has been excluded [74]. However, outside of the sphere of high‐level athletes, multiple other factors may activate the HPA axis and suppress the HPG axis in exercising men and women. Diagnosing relative energy deficiency and delineating energy deficiency from other stressors suppressing HPG axis function are very challenging [82]. Addressing barriers to better energy intake, recovery and non‐exercise stressors are often equally necessary for individuals with exercise‐associated hypogonadism.

### 3.2 Musculoskeletal Injury

Women demonstrate higher rates of lower limb musculoskeletal injuries, including more severe injuries like bone stress and anterior cruciate ligament (ACL) injuries than men when in physically arduous employment [87]. Sex differences in bone and tendon physiology underpin these differences. During puberty, 17*β*‐oestradiol inhibits periosteal bone formation and promotes endosteal bone formation in girls, whereas in men, testosterone supports continued periosteal expansion and endosteal remodelling, strengthened by greater force‐driven remodelling resulting from greater skeletal muscle mass [88]. By adulthood, men have a thicker cortex and larger cortical and total bone areas (measured at the tibia as a reference site by cross‐sectional imaging) [89].

Women experience higher rates of stress fractures (where repetitive use leads to material fatigue and disruption to microarchitecture, a common injury in sports and physically arduous occupations) [75]. High volumes and a rapid onset of training increase the risk, together with nutritional deficiencies. Oligo or amenorrhoea, particularly alongside insufficient energy intake, is associated with poor bone microarchitecture, osteopenia and increased rates of stress fractures, presumably due to the deleterious effects of calorie and oestradiol deficit on bone turnover [74]. Low endogenous oestradiol can inhibit some of the skeletal adaptations with loading, although weight‐bearing exercise may provide some protection to the deleterious effects of low oestradiol [90]. Most hormonal contraceptives do not appear to influence bone metabolism or microarchitecture [91], except depot medroxyprogesterone acetate, which reduces bone density and increases the risk of fracture [90]. Transdermal administration of 17*β*‐oestradiol improves load‐related bone microarchitecture in oligomenorrhoeic athletes [92].

Sex differences in tendon injuries are influenced by smaller tendons and greater laxity, or decreased stiffness, in women, contributing to a two to eight times higher risk of ACL rupture in female than male athletes, even after accounting for other factors like training protocol or behaviour [93]. Women synthesise and incorporate less collagen into tendons in response to exercise training than men, due to differential effects of testosterone and 17*β*‐oestradiol on collagen. Both increase collagen synthesis; 17*β*‐oestradiol decreases ligament stiffness through inhibiting lysyl oxidase and reducing collagen cross‐linking [93]. The mechanical properties of ligaments can surprisingly change within days: women demonstrate greater joint laxity (and purportedly experience more ACL injuries) during the late follicular phase, when oestrogen levels peak and progesterone are low [93]. While a stiffer ligament helps prevent injury, the relationship between tendon stiffness and muscle injury is more complex; however, a stiffer tendon supports better muscle performance [94]. Decreased tendon stiffness can reduce the propensity to some injuries: women experience fewer Achilles tendon injuries than age‐matched men, until after the menopause, when the risk becomes similar in both sexes [94].

### 3.3 Heat Illness and Exertional Hyponatraemia

Exercise is inherently inefficient, with up to 80% of energy invested in strenuous physical activity being released as heat. This thermal energy needs to be moved from the body's core to the external environment by processes under the regulation of the autonomic nervous system and various hormones. Environmental temperature and humidity are important for the effectiveness of these processes, but heat illness can occur at any ambient temperature. Unless humidity is high, most heat is divested via evaporative cooling of sweat, with peripheral vasodilatation serving to ensure circulating volume reaches eccrine sweat glands. Vasodilation also contributes to cooling through convection [95].

On average, anthropometric factors, including percent body fat and a higher body surface area: mass ratio (surface specific area), make women more prone to heat storage [96]. As noted above, progesterone reduces vasodilation, increases core temperature and reduces evaporative cooling. However, sex differences in incapacitation under heat stress, arguably the best ‘hard endpoint’ of performance degradation, do not bear out these differences [97]. Extant rates of heat illness appear significantly lower in women than in men, which perhaps reflects sex differences in behaviour [76].

Brisker catecholaminergic vasculature responses in men support continuation of exercise in the face of threatened syncope [37, 98]. This may also divert a greater proportion of cardiac output, under pressure to support skin blood flow and exercising muscles, away from the viscera and brain. This could explain the greater burden of exertional heatstroke and biochemical evidence for organ injury among men, while lesser forms of heat illness appear commoner among women [99].

Potential behavioural differences in the sexes are also indicated by a greater tendency to exertional hyponatraemia in women, with a tendency to replace water deficits at a greater rate than men possibly exacerbating constitutive hormonal predisposition to greater water retention [100].

The great majority of investigations into thermal tolerance and heat adaptation (including those pioneered by Jerome Conn, who characterised the action of aldosterone in acclimatisation among US Service personnel during and after World War 2) have been accomplished in men, and more research is required to understand how performance under heat stress is best supported in women.

### 3.4 Exogenous Steroids

Supplementation (‘doping’) with exogenous anabolic androgenic steroids (AASs) has become, unfortunately, a relatively common occurrence in male athletes and is thought to be on the increase [101] with around 50% of all adverse analytical findings in athletes’ urine per year attributed to AASs [102]. This is perhaps unsurprising given that testosterone and related derivatives can confer a very significant performance advantage through an increase in lean muscle mass and therefore strength, alongside haematopoietic benefits that improve oxygen carrying capacity.

While there is some evidence that females with endogenous hyperandrogenic states (such as Polycystic Ovary Syndrome and Congenital Adrenal Hyperplasia) may benefit from a positive influence on lean mass and muscle strength, the evidence regarding any performance advantage is very inconsistent [103]. This most likely reflects the difference between relatively minor physiological variations outside the normal range versus exogenous AASs giving supra‐physiological levels with a clear performance advantage. As such, it is also unsurprising that they have become the commonest drug of abuse by elite female athletes [104]. Probably the most compelling evidence regarding the performance enhancement of AASs is provided by documentation released after the unification of Germany that revealed the systematic doping of athletes with AASs in the German Democratic Republic between 1965 and 1989. Such use in females could be associated with an increase in shot put of 4.5−5 m and 1500 m run time improved by 7−10 s [103], the difference between being knocked out in the qualifying heats or a podium position.

This is not just an issue for elite‐level athletes but is seen in those performing at lower levels, including recreational athletes, and is a particular problem among the bodybuilding community. The use of AASs is not without risk or complication. The incidence of anabolic steroid‐induced hypogonadism (ASIH) is an increasingly common presentation [105], complicated by the deleterious effect on spermatogenesis. Users will often employ a variety of methodologies to counteract this including cyclical use of AASs to try and permit recovery of the HPG axis between episodes; the use of HCG to maintain pituitary drive and selective ER modulators (such as clomiphene) and aromatase inhibitors (such as anastrozole) to inhibit negative feedback at the hypothalamus and maintain pituitary gonadotrophin secretion [101]. The issue is becoming ever more problematic with the surge in online/private companies offering TRT to those with normal biochemical parameters in conjunction with such drugs, increasing availability.

Adverse effects are not isolated to males with females at risk of virilisation (including but not limited to alopecia, acne, hirsutism, menstrual disturbance, deepening voice, clitoromegaly and aggression) and along a spectrum to liver toxicity, including hepatic rupture, as well as a risk of sudden cardiac death [103]. As endocrinologists, we may bear witness to the pervasive evolution in society where the use of drugs is not limited to their abuse for performance enhancement, despite the risks.

## 4 Summary

Though biological sex differences can be discerned by 13 weeks of foetal life, and further pattern differences result from ‘minipuberty’ in infancy, the most striking sex differences result from the 15–20‐fold higher testosterone levels in males than in females from puberty onwards. A sex difference in athletic performance persists between women and men. Whilst the degree to which various characteristics of anatomy, physiology, metabolism and behaviour contribute to these differences, there are multiple biologically plausible mechanisms. Women are smaller than men and have a relatively higher fat mass and lower skeletal muscle mass. The skeletal muscle of men is stronger, largely as a result of a higher contribution of fast‐twitch fibres. However, this fibre proportion also contributes to the higher relative fat oxidation, at any given level of exertion, and the enhanced fatigue resistance of female muscle. Men have larger hearts, higher stroke volumes, higher haemoglobin mass and haematocrit than women, not only in absolute terms, but also when normalised to lean body mass. All these differences contribute to the higher maximal oxygen uptake (V̇O_2_ max) of men and are critical in superior endurance exercise performance.

Sex is not only fundamental to differences in performance but also in susceptibility to exercise‐associated pathology. Though women have an increased tendency to retain heat, and to begin thermoregulatory peripheral vasodilation and sweating at a higher temperature than men (a phenomenon which varies with the menstrual cycle stage), they appear in fact to be less vulnerable to severe heat illness than men. This may relate to a relatively reduced diversion of blood away from vital organs towards skeletal muscle and skin in women than in men. Women are more likely to experience hypogonadism than men because of strenuous exertion and relative energy deficit. This is frequently discernible through oligo‐ or amenorrhoea. Importantly, another feature of this condition is osteopenia, and female athletes are more susceptible to lower limb stress fractures than men conducting similar training, as indeed they are to other connective tissue injuries, such as rupture of the ACL. Regardless of sex, exercise is an essential element of human health. In women in particular, load‐bearing exercise, which is well supported by adequate energy intake, is fundamental to the maintenance of bone health and reducing the risk of osteoporosis.

## Funding

The authors have nothing to report.

## Conflicts of Interest

The authors declare no conflicts of interest.

## Data Availability

Data sharing is not applicable to this article as no data sets were generated or analysed during the current study.
